# Association of Circulating C1q/TNF-Related Protein 1 Levels with Coronary Artery Disease in Men

**DOI:** 10.1371/journal.pone.0099846

**Published:** 2014-06-19

**Authors:** Daisuke Yuasa, Koji Ohashi, Rei Shibata, Kyosuke Takeshita, Ryosuke Kikuchi, Ryotaro Takahashi, Yoshiyuki Kataoka, Megumi Miyabe, Yusuke Joki, Takahiro Kambara, Yusuke Uemura, Kazuhiro Matsuo, Satoko Hayakawa, Mizuho Hiramatsu-Ito, Masanori Ito, Nobuo Ikeda, Toyoaki Murohara, Noriyuki Ouchi

**Affiliations:** 1 Department of Cardiology, Nagoya University Graduate School of Medicine, Nagoya, Japan; 2 Department of Molecular Cardiology, Nagoya University Graduate School of Medicine, Nagoya, Japan; 3 Department of Cardiology, Chunichi Hospital, Nagoya, Japan; University of Warwick – Medical School, United Kingdom

## Abstract

**Objective:**

Obesity is a major risk factor for cardiovascular disease. Recent evidence demonstrates that dysregulation of fat-derived hormones, also known as adipokines, is linked with the pathogenesis of obesity-related disorders including coronary artery disease (CAD). Here, we investigated whether circulating level of an adipokine C1q/TNF-related protein (CTRP) 1 is associated with the prevalence of CAD.

**Methods and Results:**

Consecutive 76 male CAD patients were enrolled from inpatients that underwent coronary angiography. Sixty four healthy male subjects served as controls. Plasma CTRP1 concentration was determined by enzyme-linked immunosorbent assay. CTRP1 levels were correlated positively with systolic blood pressure (BP) and triglyceride levels, and negatively with HDL cholesterol levels in all subjects. Plasma levels of CTRP1 were significantly higher in CAD patients than in control subjects (CAD: 443.3±18.6 ng/ml, control: 307.8±21.5 ng/ml, p<0.001). Multiple logistic regression analysis with body mass index, systolic BP, glucose, total cholesterol, HDL cholesterol, triglyceride, adiponectin and CTRP1 revealed that CTRP1 levels, together with systolic BP and HDL cholesterol, correlated with CAD.

**Conclusions:**

Our data indicate the close association of high CTRP1 levels with CAD prevalence, suggesting that CTRP1 represents a novel biomarker for CAD.

## Introduction

Pandemic increase of obese subjects is a social problem in the industrialized countries. Obesity causes a number of metabolic disorders including type 2 diabetes, dyslipidemia, and hypertension, ultimately leading to the development of atherosclerotic diseases including coronary artery disease (CAD) [1]–[6]. Accumulating evidence shows that obese conditions induce the dysregulated production of adipose tissue-derived hormones, also referred to as adipokines, which considerably contributes to the pathogenesis of various obese complications [7]–[9]. A number of pro-inflammatory adipokines including tumor necrosis factor (TNF)-α are up-regulated in obese adipose tissue, and these conditions deteriorate obesity-related diseases [7], [8]. In contrast, obese states reduce the production of several anti-inflammatory adipokines including adiponectin, thereby leading to the progression of obesity-linked metabolic and vascular diseases [7], [10]–[14].

C1q/TNF-related proteins (CTRPs) were identified as paralogs of adiponectin that have the common structural domains including collagenous and globular C1q-like domains [15]. CTRP1 was identified as an adipokine that is abundantly expressed in adipose tissue [15]–[17]. CTRP1 has been reported to reduce blood glucose levels in mice [16], [18]. Overexpression of CTRP1 also improves insulin sensitivity and glucose tolerance under conditions of obesity [18]. A recent clinical study demonstrated that high levels of circulating CTRP1 are associated with metabolic syndrome [19]. Thus, these observations suggest that CTRP1 is associated with obesity-related metabolic disorders. However, nothing is known about the relationship between CTRP1 and cardiovascular disease. Here we investigated whether plasma CTRP1 levels are associated with the prevalence of CAD.

## Materials and Methods

### Study Subjects

Consecutive 76 male CAD patients were enrolled from inpatients that underwent coronary angiography at Nagoya University Hospital between 2009 and 2011. The criteria of CAD were a 75% or greater organic stenosis of at least one major coronary artery as confirmed by coronary angiogram. We excluded patients with acute myocardial infarction, congestive heart failure, hemodialysis and malignancy. Sixty four subjects were recruited from healthy subjects who visited Chunichi Hospital for a medical checkup. All control subjects have no history of CAD and medication. Diabetes mellitus was determined by criteria of World Health Organization and/or having treatment for diabetes mellitus. All patients and control subjects were Japanese and gave written informed consent. This study was approved by the ethics committee of the Nagoya University School of Medicine and Chunichi Hospital.

### Laboratory Methods

Blood samples were obtained from CAD patients and control subjects after an overnight fasting. Plasma CTRP1 levels were measured by enzyme-linked immunosorbent assay (ELISA) kit (BIO Vendor, NC, USA) for human CTRP1, and the intra-assay and inter-assay coefficients of variation were 2.6% and 9.1%, respectively (limit of detection: 6.25 ng/ml). Plasma adiponectin levels were determined by a latex turbid-metric immunoassay according to the manufacturer’s protocol (Otsuka Pharmaceutical Corporation, Tokushima, Japan). Total cholesterol, high density lipoprotein (HDL) cholesterol, low density lipoprotein (LDL) cholesterol, triglycerides, glucose, and creatinine levels were measured by standard assays. Blood pressure (BP) was measured with an appropriate arm cuff and a mercury column sphygmomanometer after at least 10 minute rest in sitting position. Body mass index (BMI) was calculated as the ratio of weight (kg) to squared height (m^2^). Estimated glomerular filtration rates (eGFR) were evaluated by circulating creatinine (Cr) levels, age and sex according to the Simplified Modification of Diet in Renal Disease equation for Japanese. The exact calculation of eGFR was performed by 194×Cr (mg/dl)^−1.094^×Age (years)^−0.287^ according to the formula in men.

### Statistical Analysis

Values were presented as mean ± standard error (SE) for continuous variables. Associations between CTRP1 and the indicated parameters were examined by simple correlation analysis. Associations between CAD and all other parameters were first examined by simple logistic regression analysis, and then evaluated by multiple logistic regression analysis using parameters selected from single analysis. We estimated the odds ratio corresponding to a 1 standard deviation increase in each measure of the indicated parameters. A value of p<0.05 was considered as statistically significant. All analyses were performed using JMP pro (version 10; SAS institute).

## Results

### Clinical Characteristics

**Figure 1:**
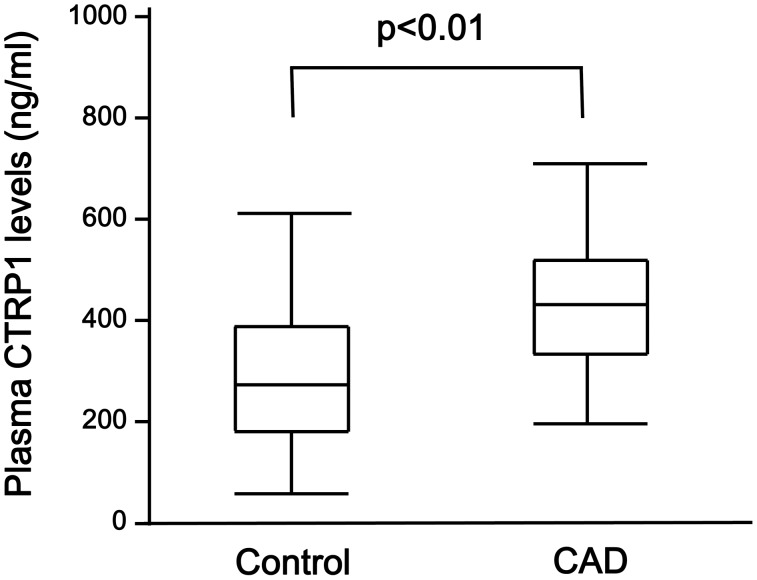
Plasma CTRP1 levels in control subjects and patients with CAD. Plasma concentration of CTRP1 in control subjects (n = 64) and CAD patients (n = 76) was measured by ELISA system.

Clinical characteristics of male CAD patients and control subjects are shown in Table 1. CAD patients had significantly higher levels of BMI, systolic BP, triglyceride, fasting glucose, prevalence of diabetes mellitus and the frequency of medication use than control subjects. Total cholesterol, HDL cholesterol and adiponectin levels were lower in CAD patients than in control subjects. Plasma CTRP1 levels were significantly higher in CAD patients than in control subjects (Figure 1). There were no significant differences in age, the frequency of smokers, diastolic BP, LDL cholesterol, creatinine and eGFR between two groups (Table 1).

**Table 1:** Clinical characteristics of control subjects and CAD patients.

Characteristics	Control (n = 64)	CAD (n = 76)	P value
**Age (years)**	61.5±0.6	63.7±1.0	0.065
**BMI (kg/m^2^)**	22.9±0.3	24.5±0.4	<0.01
**Smoking (%)**	37.5	31.6	0.462
**Systolic BP (mmHg)**	115.3±2.2	124.5±1.8	<0.01
**Diastolic BP (mmHg)**	71.9±1.4	70.5±1.1	0.431
**Glucose (mg/dl)**	102.9±1.4	108.9±2.3	<0.05
**Diabetes (%)**	9.4	40.8	<0.001
**Total cholesterol (mg/dl)**	186.0±3.8	174.9±3.7	<0.05
**LDL cholesterol (mg/dl)**	106.6±3.1	104.0±3.2	0.557
**HDL cholesterol (mg/dl)**	57.1±1.7	45.0±1.4	<0.001
**Triglyceride (mg/dl)**	104.3±8.0	126.6±7.2	<0.05
**Creatinine (mg/dl)**	0.88±0.02	0.90±0.05	0.670
**eGFR (ml/min/1.73 m^2^)**	70.6±1.6	72.4±2.2	0.514
**Adiponectin (µg/ml)**	6.54±0.51	5.30±0.21	<0.05
**CTRP1 (ng/ml)**	307.8±21.5	443.3±18.6	<0.001
**Medication (%)**			
**Antihypertensive**	0	66.7	<0.001
**Anti-diabetic**	0	26.9	<0.001
**Cholesterol lowering**	0	68.0	<0.001

Data are presented as mean ± SE.

BMI; body mass index, BP; blood pressure, LDL; low density lipoprotein.

HDL; high density lipoprotein, eGFR; estimated glomerular filtration rate.

CTRP1; C1q/TNF-related protein-1.

### Correlation between CTRP1 and Clinical Parameters

**Figure 2:**
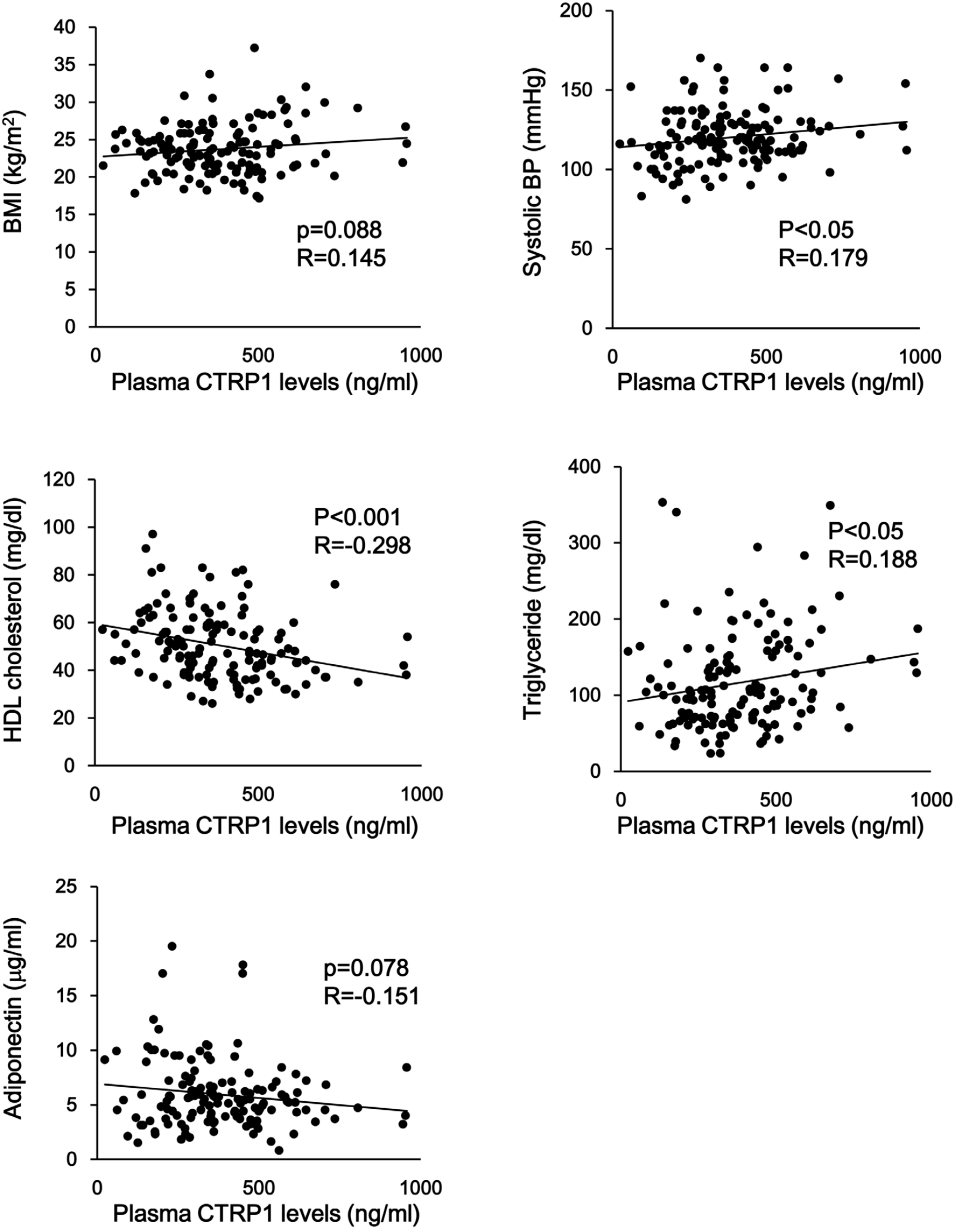
Correlation between plasma CTRP1 levels and conventional risk factors. Correlation analyses between plasma CTRP1 levels and risk factors, including body mass index (BMI), systolic blood pressure (BP), high density lipoprotein (HDL) cholesterol, triglyceride and adiponectin levels were evaluated.

We next assessed the relationship between plasma levels of CTRP1 and clinical parameters in all subjects. Plasma CTRP1 levels were correlated positively with systolic BP and triglyceride levels and negatively with HDL cholesterol levels in all subjects (Figure 2) (Table 2). In addition, plasma CTRP1 levels tended to correlate positively with BMI and negatively with adiponectin levels in all subjects (Figure 2) (Table 2). In contrast, plasma CTRP1 levels did not correlate with age, diastolic BP, glucose, total cholesterol, LDL-cholesterol, Cr and eGFR in all subjects (Table 2). In CAD patients, plasma CTRP1 levels were correlated positively with triglyceride levels and negatively with HDL cholesterol levels (Table 2). Plasma CTRP1 levels tended to correlate positively with BMI and negatively with adiponectin levels in CAD patients. In contrast, no significant correlation was observed in control subjects between CTRP1 levels and any clinical parameters.

**Table 2:** Correlation between plasma CTRP1 levels and clinical parameters.

	All subjects (n = 140)		Control (n = 64)		CAD (n = 76)	
	Coefficient	P value	Coefficient	P value	Coefficient	P value
Age (years)	0.047	0.586	0.082	0.520	–0.067	0.569
BMI (kg/m^2^)	0.145	0.088	–0.182	0.150	0.211	0.069
Systolic BP (mmHg)	0.179	<0.05	0.006	0.961	0.166	0.155
Diastolic BP (mmHg)	0.007	0.931	–0.052	0.684	0.121	0.302
Glucose (mg/dl)	0.105	0.220	–0.067	0.597	0.093	0.429
Total cholesterol (mg/dl)	–0.146	0.087	–0.141	0.266	–0.041	0.730
LDL cholesterol (mg/dl)	–0.008	0.356	–0.145	0.253	0.001	0.995
HDL cholesterol (mg/dl)	–0.298	<0.001	–0.083	0.512	–0.253	<0.05
Triglyceride (mg/dl)	0.188	<0.05	–0.028	0.826	0.287	<0.05
Creatinine (mg/dl)	0.018	0.835	0.020	0.874	0.002	0.984
eGFR (ml/min/1.73 m^2^)	–0.005	0.957	–0.027	0.832	0.010	0.934
Adiponectin (µg/ml)	–0.151	0.078	–0.028	0.828	–0.208	0.073

BMI; body mass index, BP; blood pressure, LDL; low density lipoprotein.

HDL; high density lipoprotein, eGFR; estimated glomerular filtration rate.

### Relationship of CTRP1 with CAD

To determine the relationship between CTRP1 and CAD prevalence, single and multiple logistic regression analyses were performed. In single logistic regression analysis, BMI, systolic BP, fasting glucose, total cholesterol, HDL cholesterol, triglyceride, adiponectin and CTRP1 were significantly associated with CAD (Table 3). Multiple logistic regression analysis with BMI, systolic BP, glucose, total cholesterol, HDL cholesterol, triglyceride, adiponectin and CTRP1 demonstrated that systolic BP, HDL cholesterol and CTRP1 significantly associated with CAD.

**Table 3:** Association with CAD.

		Single		Multiple	
	1SD	OR (95%CI)	P value	OR (95%CI)	P value
Age (years)	7.1	1.047(0.998–1.101)	0.060		
BMI (kg/m^2^)	3.3	1.181(1.056–1.337)	<0.01	1.060(0.920–1.237)	0.434
Smoking (%)		1.300(0.645–2.627)	0.463		
Systolic BP (mmHg)	17.2	1.035(1.013–1.060)	<0.01	1.036(1.010–1.066)	<0.05
Diastolic BP (mmHg)	10.6	0.987(0.956–1.019)	0.429		
Glucose (mg/dl)	16.7	1.024(1.003–1.047)	<0.05	1.006(0.979–1.036)	0.650
Total cholesterol (mg/dl)	31.7	0.989(0.977–0.999)	<0.05	0.994(0.979–1.009)	0.423
LDL cholesterol (mg/dl)	26.7	0.996(0.984–1.009)	0.556		
HDL cholesterol (mg/dl)	14.3	0.931(0.901–0.958)	<0.001	0.951(0.914–0.985)	<0.01
Triglyceride (mg/dl)	64.0	1.006(1.000–1.012)	<0.05	1.001(0.994–1.009)	0.800
Creatinine (mg/dl)	0.31	1.282(0.417–4.946)	0.663		
eGFR (ml/min/1.73 m^2^)	16.3	1.007(0.986–1.028)	0.513		
Adiponectin (µg/ml)	3.11	0.871(0.765–0.977)	<0.05	1.002(0.854–1.160)	0.981
CTRP1 (ng/ml)	178.8	1.005(1.003–1.008)	<0.001	1.004(1.001–1.007)	<0.01

The odds ratios corresponding to a 1 SD increase in each measure of the indicated parameters were estimated. SD; standard deviation, CI; confidence intervals, BMI; body mass index, BP; blood pressure, LDL; low density lipoprotein, HDL; high density lipoprotein, eGFR; estimated glomerular filtration rate, CTRP1; C1q/TNF-related protein 1.

## Discussion

This study for the first time demonstrates that increased levels of circulating CTRP1 are associated with the prevalence of CAD. High levels of CTRP1 have been shown to associate with the metabolic syndrome [19]. It has also been shown that CTRP1 levels are increased in hypertensive patients [20]. In agreement with these findings, our data indicate that plasma CTRP1 levels associate positively with systolic BP and triglyceride levels and negatively with HDL cholesterol levels in all subjects. Because both metabolic dysfunction and high blood pressure are causally linked with the development of CAD [4]–[6], it is conceivable that these disease conditions could mediate the association between CTRP1 and CAD prevalence. However, we note that CTRP1 levels are predictive of CAD, independent of conventional risk factors for CAD. Therefore, CTRP1 may be a useful biomarker for evaluation of cardiovascular risk.

The functional role of CTRP1 in the cardiovascular system is completely unknown. It has been shown that CTRP1 improves glucose metabolism and reduces adiposity under conditions of over-nutrition [18]. It has also been reported that CTRP1 exerts an anti-platelet thrombotic activity in a model of vascular injury, indicating the possibility of the vascular effects of CTRP1 [21]. Taken together with the present findings, these results suggest that CTRP1 not only represents a cardiovascular biomarker but also functions as an adipokine that regulates metabolic and vascular disorders. However, future researches are required to elucidate the impact of CTRP1 on cardiovascular homeostasis.

Obese complications are characterized by chronic low-grade inflammation [7], [22], [23]. It has been reported that the pro-inflammatory mediators including TNF-α and IL1β are involved in the induction of CTRP1 expression in adipose tissue of Sprague-Dawley rats [17]. In this regard, obese Zucker diabetic fatty rats show high expression levels of CTRP1 in adipose tissue [17]. Similarly, obese ob/ob mice exhibit increased expression of CTRP1 in fat tissue [16]. On the other hand, CTRP1 levels are reported to positively correlate with BMI in subjects with metabolic syndrome, but not in healthy subjects [19]. In the present study, plasma concentration of CTRP1 tended to positively correlate with BMI in CAD patients and all subjects. In addition, no significant association was observed in control subjects between CTRP1 levels and BMI. Thus, future clinical studies will be needed to clarify the relationship of CTRP1 with adiposity and inflammatory states in a larger population. Furthermore, it is well-established that circulating levels of anti-inflammatory adipokine adiponectin are reduced by obesity [24]. Interestingly, adiponectin deficient mice exhibit increased levels of CTRP1 compared with control mice, indicating the negative association between adiponectin and CTRP1 levels [16]. However, it has been shown that CTRP1 does not correlate with adiponectin levels in healthy individuals and subjects with metabolic syndrome [19]. Consistently, our data showed that no significant correlation is observed between CTRP1 and adiponectin levels in healthy controls, CAD patients and all subjects. Although it is plausible that CTRP1 may compensate for reduced adiponectin concentration in obese states, this assumption will require future investigation.

### Conclusion

In summary, the current study documents that elevated level of plasma CTRP1 is a novel indicator of CAD prevalence. Thus, measurement of circulating CTRP1 concentrations may be valuable for assessment of cardiovascular risk.
